# Open-Source System for Real-Time Functional Assessment of In Vitro Filtration Barriers

**DOI:** 10.1007/s10439-023-03378-9

**Published:** 2023-10-29

**Authors:** Tess K. Fallon, Merve Zuvin, Alan D. Stern, Nanditha Anandakrishnan, Ilse S. Daehn, Evren U. Azeloglu

**Affiliations:** 1 Barbara T. Murphy Division of Nephrology, Icahn School of Medicine at Mount Sinai, One Gustave L. Levy Place, Box 1243, New York, NY 10029 USA; 2 Pharmacological Sciences, Icahn School of Medicine at Mount Sinai, One Gustave L. Levy Place, Box 1243, New York, NY 10029 USA; 3 Department of Electrical Engineering, Columbia University, 500 W. 120th St, New York, NY 10027 USA

**Keywords:** Open science, Fluorescence sensor, Glomerulus, Functional assay, Microfluidics, Organ-on-chip, Kidney-on-chip

## Abstract

The integrity of the barrier between blood and the selective filtrate of solutes is important for homeostasis and its disruption contributes to many diseases. Microphysiological systems that incorporate synthetic or natural membranes with human cells can mimic biological filtration barriers, such as the glomerular filtration barrier in the kidney, and they can readily be used to study cellular filtration processes as well as drug effects and interactions. We present an affordable, open-source platform for the real-time monitoring of functional filtration status in engineered microphysiological systems. Using readily available components, our assay can linearly detect real-time concentrations of two target molecules, FITC-labeled inulin and Texas Red-labeled human-serum albumin, within clinically relevant ranges, and it can be easily modified for different target molecules of varying sizes and tags. We demonstrate the platform’s ability to determine the concentration of our target molecules automatically and consistently. We show through an acellular context that the platform enables real-time tracking of size-dependent diffusion with minimal fluid volume loss and without manual extraction of media, making it suitable for continuous operational monitoring of filtration status in microphysiological system applications. The platform’s affordability and integrability with microphysiological systems make it ideal for many precision medicine applications, including evaluation of drug nephrotoxicity and other forms of drug discovery.

**Supplementary Information:**

The online version of this article contains supplementary material available 10.1007/s10439-023-03378-9.

## Introduction

Precision medicine aims to develop patient-derived tissue analogs that enable accurate high-throughput drug screening and discovery. Two-dimensional (2D) cell culture assays used to model human tissue function are often not sufficiently translatable into the clinic or even readily recapitulated in animal models. In contrast, three-dimensional (3D) microphysiological or organ-on-a-chip (OOC) systems have been shown to more accurately recapitulate human physiological conditions [1–4]. For example, Homan et al. showed that vascularization of kidney organoids is possible when cultured on-chip under flow, and these organoids were shown to enable in vitro culture of more mature podocytes compared to earlier 2D systems [5]. t’Hart and Yildiz et al. also demonstrate how co-culture and crosstalk between glomerular cell types within a glomerulus-on-chip positively affect barrier function, cell morphology, and transcriptional phenotype [6]. Similarly, Hirastuka et al. demonstrated that 3D kidney organoids experiencing flow can reveal autosomal recessive polycystic kidney disease mechanisms previously undiscovered in static or 2D in vitro models [1].

Another benefit of OOCs is that they can comprise multiple channels, which enable co-culture of different cell lines [7, 8]. Cellular co-culture presents an opportunity to recapitulate biological barriers, such as the blood–brain barrier, the lung-airway barrier, endothelial barriers of the gut, and the glomerular filtration barrier, in OOC devices [7, 9–14]. Many of these biological barriers are of great pharmacological interest and can be useful for exploring pathologies and drug delivery. For example, Aceves et al. demonstrate how 3D tubules-on-chip exhibit increased drug intake compared to 2D culture and highlight the tubule-on-chip’s relevance for drug screening and discovery [15]. Modeling the glomerular filter offers opportunities for various research applications and drug screening that would impact a growing number of people suffering from progressive kidney disease worldwide. The glomerular filtration barrier (GFB) is a highly specialized interface responsible for blood filtration that is charge and size selective [16]. Through co-culture of human induced pluripotent stem cell-derived podocytes and human glomerular microvascular endothelial cells, Musah et al. demonstrated the successful recapitulation of the selective filtering properties of the GFB via inulin-albumin clearance measurements and responses to nephrotoxic injury [7]. Petrosyan et al. also showed how a glomerulus-on-a-chip platform can be used to examine autoimmune-induced kidney damage [17]. Additionally, Zhou et al. demonstrated an in vitro model of hypertensive nephropathy using a similar organ-on-a-chip system by co-culturing mouse glomerular endothelial cells and podocytes. Their model showed that increasing capillary flowrates (i.e., hypertension) compromised the glomerular membrane integrity [18].

Due to their promise of more accurately modeling human physiological function and disease, OOCs have been proposed as efficient drug-screening platforms [19]. To maximize throughput of an OOC in drug-screening applications, the OOCs must be user-friendly and easy to make. Polydimethylsiloxane (PDMS), which was used in prior GFB-on-chips, requires a cleanroom to use and therefore is not optimal for rapid prototyping [6, 7, 17]. Additionally, PDMS readily absorbs small molecules [20], which can be detrimental to drug perfusion, potential functional assays, and many other applications necessary in the context of personalized medicine. In contrast, poly(methyl methacrylate), PMMA, is extensively used in microfluidic applications as a more user-friendly alternative to PDMS. Such an OOC was reported by Hosic et al. [21]; their bi- and tri-layer chips built from PMMA and polycarbonate track-etched (PCTE) membranes were integrated to build a multi-tissue gut-on-chip that was used to compare organoid and multi-tissue on-chip models of the gut epithelia. In this study, we adopt a modified version of this design to fabricate a glomerulus-on-chip and integrate it with a custom-built fluorescent sensor for real-time assessment of filtration function.

Malfunction of the selective filtration function of the GFB is associated with acute kidney injury (AKI) and chronic kidney disease (CKD). Two ways to detect and evaluate AKI and CKD are functional assessments that examine glomerular filtration rate (GFR) and urine albumin concentration [22–24]. It is vital for an on-chip GFB to have the ability to track the selective filtration status of the tissue over time, and integrated and non-invasive sensing for in vitro microphysiological platforms are central to their widespread adoption [25]. Current methods for measuring key molecule concentrations in OOC applications use absorbance or enzyme-linked immunosorbent assay (ELISA) to determine the presence of biomarkers [17]. Absorbance measurements are not generally physiologically applicable and limited since quantitative evaluation of more than one molecule is challenging. Non-invasive methods implement regenerating electrochemical or physical sensors within the microfluidic device to track target molecules. Regeneration of sensors takes up to four hours, limiting the ability to continuously measure biomarkers within the regeneration time frame using a single chip. Additionally, if there is a sudden increase in biomarker levels, sensors can saturate and values above the saturation range could be missed [26]. To broaden the application and evaluate the performance of these OOC model systems, tracking the presence of inulin, albumin, or other functionally relevant molecules in real time would be relevant for glomerular filtration barrier OOC models, and the system would need to be user-friendly, accessible, and affordable.

Here, we developed a real-time fluorescence-based sensing platform for assessing the performance of in vitro filtration barriers. This report gives detailed instructions on building a functional assay for analysis of diffusion of biological molecules across a filtration layer that mimics the glomerular filtration barrier, such as a glomerulus-on-chip, in real time. Given the recognized fundamental importance of the glomerular filtration barrier in health and disease settings, this OOC system provides a tool for studying barrier function and estimating the filtration of molecules through a cellular barrier across time without the need to continuously remove and sample liquid from the OOC. Because our device consists of custom parts fabricated using additive manufacturing, laser-cut acrylic, and electronic components that are widely available, this system is affordable, simple to build, and easy to use, and it is made to fit a variety of engineered barrier systems, thus offering the opportunity to expand access to precision medicine projects in underprivileged research settings.

## Materials and Methods

### Preparation of Solutions for Calibration

A stock solution of inulin-FITC (3 mg/mL) and HSA-Texas Red (5 mg/mL) was prepared in 1XPBS. Pre-conjugated inulin-FITC (Sigma Aldrich, #f3272) is dissolved in 1XPBS in the desired stock concentration of 3 mg per 1 mL of PBS. The dye:inulin ratio was 1,000:1 according to the manufacturer. The solution was placed on a hot plate at 37 °C with a magnetic stirrer until the inulin-FITC powder was fully dissolved. Prior to experimental usage, the inulin-FITC stock was placed on a shaker for 15–30 min to eliminate any solute precipitation.

Texas Red dye (Thermo-Fisher, #T6134) was conjugated to human-serum albumin (Sigma Aldrich, #a1653) with a molecular ratio of protein: dye of 7:1. First, a 0.1 M bicarbonate solution was prepared by dissolving 2.2 g of anhydrous sodium carbonate (Sigma Aldrich, #PHR1948) in 100 mL of deionized water, and 1.68 g of sodium bicarbonate (Fisher Scientific, #S78284) in another 100 mL of deionized water. 4 mL of the carbonate solution was combined with 46 mL of the bicarbonate solution in a large container, and the volume was brought to 400 mL with 250 mL of deionized water. 2.5 mg of protein was dissolved in 500 µL of the bicarbonate solution and left on a shaker until the protein was completely dissolved (1-2 hours). A small tube was wrapped in aluminum foil, and 0.2 mg of Texas Red Dye (Thermo-Fisher, #T6134) was dissolved in 20 µL of dimethylformamide (Fisher Scientific, #FI-05-1105) immediately before conjugation. The appropriate volume for the mixture of a 7:1 molecular ratio between the dye and the protein was calculated based on their respective molecular weights. HSA has a molecular weight of 68,000 Da and the dye has a molecular weight of 817 Da, and 11.87 µg of albumin was required for each µg of dye. Using the concentrations from the previous steps, 80 µL of the dissolved dye was added per 2 mL of dissolved HSA.

The protein solution was wrapped in foil and stirred while slowly adding the reactive dye solution. The reaction was incubated for about an hour with continuous stirring. Afterward, the tube was wrapped in foil and the reaction was incubated at room temperature for approximately an hour with continuous stirring using a shaker.

An 800 mL of the same bicarbonate solution was prepared, and dialysis cassettes (Thermo Scientific, #66333) were labeled. The protein-dye solution was transferred into the dialysis cassette(s) according to the manufacturer’s instructions. The cassettes were then placed in a beaker with at least 800 mL of the bicarbonate solution. The beaker was covered with foil and stored at 4 °C overnight. After dialyzing, the labeled proteins were aliquoted and stored at 4 °C. The sensor was calibrated with serial dilutions of dye-conjugated proteins from 0.0001 to 5 mg/mL.

### Pixel Intensity to Concentration Calibration

Varying concentrations of each target molecule solutions were prepared via serial dilution as previously described. Prior to calibration, an illumination mask was determined and applied to counteract any unevenness in illumination profile. To calibrate the sensor to read both the capillary and filtrate imaging squares, various concentrations of each target molecule were loaded into both channels and the pixel intensity was measured in both. The Python script sensor.py (Online Resource 2), was used to acquire pixel intensities for calibration. The intensity values at the capillary and filtrate channels were measured at least three times and all three values were used to curve-fit the calibration.

Pixel intensity values at each concentration were fit to a standard four-parameter non-linear regression equation using Prism™:

1 $$F\left(x\right)=d+\frac{a-d}{1+\frac{x}{c}^{b}}$$

where *F(x)* is the pixel intensity (AU), *x* is the solution concentration (mg/mL), *a* is the minimum asymptote (AU), *b* is the Hill’s Slope (AU/mg/mL), *c* is the IC50 value (mg/mL), and *d* is the maximum asymptote (AU).

Unevenness in LED illumination was corrected using a normalized empirical multiplier across all pixels. At an intermediate concentration of both dyes, three images were taken to assess the intensity values at each pixel. The multiplier was determined for each pixel to normalize all pixels at the same concentration and was applied as a mask to each image collected.

The sensitivity regions of the sensor for each target molecule were estimated via reverse mapping the maximum and minimum pixel intensities of the 95% confidence intervals for the minimum and maximum asymptotes, respectively, using the equation determined from the four-parameter regression. Error was estimated by reverse mapping the standard error of the regression into a concentration value.

### Chip Fabrication and Assembly

The filtrate and capillary channels are made from 1/16” PMMA (McMaster-Carr, #8560K17). The 3 mm filtrate channel is made of two layers taped together. The fluidic ports are cut to fit the fluidic connections (Fisher Scientific, #NC0883054) and are cut from 3/16”-thick PMMA (McMaster-Carr, #8560K211). The layers of the chip were bonded using 3 M tape (3 M, #3M155985-ND). Between the two channels, we place a 20-µm-thick PCTE membrane with pores of 1 µm diameter (Cytiva, #7091-4710). We have chosen this synthetic membrane over several other potential candidates as it is optically clear and biocompatible, and it readily allows free diffusion of biomolecules. The diameter of these pores is significantly larger than our largest target molecule, HSA, which has a diameter of approximately 2.74 nm in solution [27].

Laser cutting was done with a Thunder Laser Nova24. 1/16’’ PMMA sheets were laser cut with double-sided adhesive tapes at 70% max. power at 60 cm/s and the 3/16’’ sheets were cut at 90% power at 25 cm/s (Note: laser cutter settings might vary depending on the model). To counteract the potential tape alignment issues, the tape was bound to the PMMA prior to laser cutting and bubbles were pushed out of the tape layer using a ruler. After laser cutting, layers were adhered together by aligning the four fluidic ports on each layer using an in-house alignment tool, comprising of corner poles, and pressing them together manually. Between the top and bottom channels, a 1 µm pore-sized, the 20-µm-thick PCTE membrane was placed between the two channel layers, which were cut to confluently cover the middle channel of the chip but not the fluidic ports. Prior to applying the top layer of thicker PMMA, air plasma was applied to both sides of the membrane-attached chip. The 11/16” fluidic ports were threaded to fit the fluidic connections (Fisher Scientific, #NC0883054). After threading, the thicker layer was placed atop the channel and manually pressed together. The conjoined chip was adhered with the same tape onto a microscope slide.

The assembled chip must have ample pressure applied to prevent leakage. To ensure sufficient bonding, the assembled chip was pressed onto a 65 °C hot plate with the weight of about 5 pounds atop the chip for approximately one hour. Then, the assembled chips were kept in a vacuum chamber overnight. The following day, structural submersible sealant (McMaster-Carr, #67015A42,) was applied to the sides of the chips and was cured for 24 hours. Prior to attachment to fluidic pumps, Teflon tape (Fisher Scientific, #22163741) was applied to the fittings to prevent leakage from the inlet and outlet ports. Without ample pressure, heat bonding, external sealant, proper alignment, and Teflon tape at the fluidics ports, leakage out of the three PMMA layers and through the fluidic ports can pose issues.

### Leak Testing

Assembled chips were subjected to leak testing prior to usage. For our purposes, the chip was first tested under continuous perfusion by a Cole Parmer peristaltic pump (MasterFlex, #78018-42) for 2-3 hours in both channels. Liquid was stored in media reservoirs (Nordson EFD, #7012136) and sealed with adapter assemblies (Nordson EFD, #7012338). Barbed PC connectors were used to connect the tubing (Puri-Flex, #96419-13) to the chip. After passing the initial leak tests, the chips underwent another 48-72 hours of testing prior to flow experiments or cell seeding. All leak tests were performed at our maximal experimental flowrate of 100 µl/min to determine the durability of the chip under comparable conditions. After leak testing, the chip was dried and stored for experiments.

### Sample Illumination

The LEDs were turned on one at a time to prevent crosstalk. Prior to sample input, a background image was collected for each LED and the pixel values of that image were subtracted from all subsequent images. First, the 490 nm (cyan) LED was turned on, exciting FITC. The intensity value was collected, and the cyan LED was turned off. The 590 nm (amber) LED was turned on, exciting Texas Red. The intensity value was collected. Each “cycle” of intensity collection was a single time point, and the maximum time resolution for our given equipment setup was limited to approximately seven seconds.

### Diffusion Percentage Calculations

Diffusion percentage was calculated using:

2 $$Diff\%=\frac{{C}_{f,filtrate}}{{C}_{i,capillary}}\times100$$

where *C*_*f,filtrate*_ is the final concentration in the filtrate channel and *C*_*i,capillary*_ is the initial concentration in the capillary channel.

### Shear Stress Calculations

To assess the diffusion percentage across the PCTE membrane at varying shear stresses, approximate shear stress at the membrane was calculated at various flowrates and diffusion percentage was assessed. Shear stress was approximated using

3 $$\tau =\frac{6Q\mu }{w{h}^{2}}$$

where *τ* is the total shear stress (dynes/cm^2^) experienced at the polycarbonate membrane, *Q* is the flowrate (mL/s), *µ* is the viscosity of water at 37 °C (0.006913 poise), *w* is the channel width, and *h* is the channel height [21].

### Assessment of Flowrate on Diffusion Percentage

A cell-free context was used to examine changes in steady-state diffusion percentages due to alterations in volumetric flowrate. Five mL of 1 mg/mL inulin-FITC solution was perfused in the capillary channel. 2.5 mL of 1XPBS was perfused through the filtrate channel. At 100 µL/min, the system’s diffusion was brought to an equilibrium (around 20 min). Without disturbing the microfluidic setup, the volumetric flowrate was altered and the diffusion percentage of inulin-FITC was examined across time. Between each flowrate test, the microfluidic device was flushed with 1X PBS to remove fluorescence from previous runs. Starting with a perfusion rate of 100 µL/min, flowrate was decreased incrementally to 50 µL/min, 25 µL/min, and 8 µL/min. For these experiments, the sensor sampling rate was once every 2 min.

### Fluid Dynamic and Diffusion Predictions

Based on the fluid dynamics of the microfluidic device, it was predicted that diffusion percentage would be impacted by both the concentration gradient applied across the membrane and flowrate in the capillary channel. Two separate approaches were used to estimate predictions for complex diffusion behavior: estimates of steady-state equilibrium concentrations in both channels and transient behavior for the filtrate concentrations responding to changes in fluid dynamics.

Darcy’s Law was used to approximate the steady-state flux-per-unit-area ($\frac{\frac{{\mu m}^{3}}{\mu {m}^{2}}}{s}$) across the semiporous membrane, which can be described through

4 $$j=\frac{\kappa }{\mu h}\Delta P$$

where *κ* is the permeability constant of the target molecule through the PCTE membrane (µm^2^), *ΔP* is the pressure differential across the membrane (Pa), *μ* is the fluid viscosity (Pa*s), and *h* is the membrane thickness (µm) [28]. Permeability constant is a physical constant dependent on both membrane and molecule properties [29, 30]. It was estimated by perfusing 5 mL of 0.1 mg/mL inulin-FITC and HSA-Texas Red solution into the capillary channel for 30 min at 100 µL/min and measuring the filtrate channel concentration after the 30 min using a SpectraMax® M3 Multi-Mode Microplate Reader (Molecular Devices, LLC, San Jose, CA, USA), which then could be used to extrapolate the permeability of the molecules using Darcy’s Law and the microfluidic device’s fluidic properties. *κ* was estimated to be 2.01E−4 and 9.67E−4 µm^2^ for inulin and albumin, respectively.

Pressure differential was approximated as the sum of the hydrostatic and osmotic pressures [31]

5 $$\Delta P={\pi }_{hs}+{\pi }_{osm}$$

The hydrostatic pressure was estimated using Bernoulli’s Law:

6 $${\pi }_{hydrostatic}=0.1(\rho gh-\frac{1}{2}\rho {v}^{2})[Pa]$$

and the osmotic pressure was calculated using [32]

7 $${\pi }_{osm}=MRT [atm]$$

where *M* is the molarity of the capillary channel (mol/L); *R* is the universal gas constant (0.08206 atm*L/mol*K); *T* is the temperature in Kelvin; *ρ* is the fluid density (g/cm^3^); *g* is the acceleration due to gravity (cm/s^2^); *h* is the capillary channel height (cm); and *υ* is the linear velocity of the fluid flow (cm/s). Prior to application to Eq. (4), all pressures were converted to Pascals.

The total fluidic flux through the membrane was calculated by multiplying the flux-per-unit area by the exposed area of the channel membrane, expressed as:

8 $$J=jA*\frac{{10}^{-12}\,c{m}^{3}}{{\mu m}^{3}}*\frac{1\,mL}{c{m}^{3}}=\frac{jA*{10}^{-12}\,\left(mL\right)}{s}$$

where *A* is the exposed channel area (cm^2^).

The flux of the target molecule across the membrane was estimated by multiplying the fluidic flux by the concentration of the capillary solution

9 $$\frac{J\, mL}{s}*\frac{{C}_{0}\, mg}{mL}=\frac{J*{C}_{0 }\,mg}{s}$$

where C_0_ is the initial concentration of the capillary solution (mg/mL).

The final predicted concentration in the filtrate segment at steady-state equilibrium was determined by computing how many seconds the fluid was exposed to the membrane through

10 $$seconds=\frac{Q\,mL}{s}*\frac{channel}{{V}_{c}\,mL }=\frac{Q\,channels}{{V}_{c}\,seconds}\to \frac{\frac{{V}_{c}}{Q}\,seconds}{channel}$$

where *V*_*c*_ is the capillary channel volume (mL) and *Q* is the volumetric flowrate (mL/s). Combining the flux from Eq. (9) and the membrane exposure time from Eq. (10), the estimated final concentration in the filtrate channel at steady state is given by

11 $$\frac{J{C}_{0}\, mg}{s}*\frac{{V}_{c}\, s}{Q\, channel}*\frac{channel}{{V}_{f}\, mL}=J\frac{{V}_{c}}{{QV}_{f}}\frac{mg}{mL}$$

where *V*_*f*_ is the volume of the filtrate channel (mL).

The transient behavior of the filtrate concentration’s response to a change in the fluidic system is complex, but it can be solved using Fick’s Second Law, with zero internal reactions [30]. To determine the theoretical time-dependent behavior of the filtrering mass, the system was treated as a diffusion dominated mass-transport system [33]. Recently, a lumped parameter model was shown to model such mass-transport phenomena in multilayer systems [34], whereby the ordinary differential equations for conservation of mass can be solved using an electrical analog model. In our system, the capillary channel was treated as a constant source of mass, and the filtrate channel was treated as an acceptor of mass with resistive and capacitive elements and a characteristic time constant *τ*. As such, the theoretical transient response of the filtrate concentration can be predicted using the equation

12 $${c}_{f}\left(t\right)=a+b\left(1-{e}^{-\frac{t}{\uptau }}\right)$$

where *c*_*f*_*(t)* is the filtrate concentration, *a* is the starting equilibrium concentration value, predicted via methods outlined above, *b* is an equilibrium value such that *a + b* is the final equilibrium value also predicted via methods outlined above, and *τ* is the time constant of the molecule moving through the system, which was estimated based on prior literature as 120 s [35].

### Real-Time Capability Testing

To assess the sensor’s ability to track changes in the system in real time, the sensor was set to sample every 15 seconds. The microfluidic device was equilibrated with 4 mL of 0.1 mg/mL inulin-FITC solution in the capillary channel and 2 mL of 1XPBS in the filtrate channel, both at 8 µL/min. A Y-connector (Harvard Apparatus, Barbed Fitting Kit, #72-1410) was placed close to the inlet of the capillary channel and was kept closed with a surgical clamp (Fisher Scientific, #22-079-749) until the system equilibrated (10-20 mins). After equilibration, 1.5 mL of a 0.5 mg/mL inulin-FITC solution was injected into the capillary channel and allowed to equilibrate. 1.5 mL of 1XPBS was then injected into the capillary channel. This process was repeated three times.

An additional experiment was performed to more closely examine the behavior of the filtrate response to changes in concentration gradient. The sensor was set to sample every 15 seconds. The system was equilibrated at a baseline concentration of 0.1 mg/mL. Four mL of the 0.1 mg/mL solution was perfused at 8 µL/min through the capillary channel and 2 mL of 1XPBS was perfused through the filtrate channel. After the system equilibrium, 1.5 mL of 0.2 mg/mL inulin-FITC solution was injected into the capillary inlet via the Y-connector. The system was again equilibrated, and a 1.5 mL injection of 0.4 mg/mL was injected into the capillary inlet. The experiment was repeated three times, and to accurate averaging across different trials, all three vectors were aligned at the points of injection.

### Cell Culture and Cell Seeding

An immortalized human podocyte cell line stably expressing mScarlet-LifeAct [36] was cultured in RPMI (Life Technologies, #11875119) medium containing 10% fetal bovine serum (FBS), (Milipore Sigma, #26140079), 1% insulin–transferrin–selenium (ITS), (Life Technologies, #51300044), and 1% penicillin–streptomycin (PS) (Life Technologies, #15160122) under permissive conditions at 33 °C for proliferation. Prior to cell seeding in the chip, mScarlet podocytes were differentiated for 10 days under non-permissive conditions at 37 °C. Human umbilical vein endothelial cells (HUVECs) stably expressing mClover-LifeAct were cultured in Endothelial Cell Growth Media with growth supplement (R&D Systems Inc, #CCM027) and 1% PS at 37 °C. The chips were UV sterilized for 30 min; then both sides were coated with 1% fibronectin (Thermo-Fisher, #33016015). After 1.5 hours, more fibronectin was added to the filtrate segment, and the chip was flipped, and incubated for an additional 1.5 hours in the laminar flow hood. The chips were washed with media following removal of fibronectin. Differentiated podocytes were trypsinized (Fisher Scientific, #25300054) and 150,000 cells were seeded to the filtrate segment of the chip. After a quick examination under an inverted microscope to confirm the presence of cells in the channel, the chip was immediately flipped and incubated at 37 °C with media (RPMI) for three hours to allow cells to adhere to the bottom side of the membrane. Endothelial cells were trypsinized and 400,000 cells were seeded to the top side of the membrane supplemented with media. A dish containing PBS was incubated along with the chip to maintain local humidity. After overnight incubation, cell adherence was confirmed on an inverted microscope, and the chip was connected to a peristaltic pump in a sterile environment and then transferred back to the 37 °C incubator to initiate flow. Media reservoirs (Nordson EFD, #7012136) containing RPMI and endothelial growth media were connected to the pump and the chip to allow for continuous media perfusion in a closed loop system, at a 100 µl/min flowrate. Cell monolayers were imaged after 48 hours using Olympus FV3000 confocal microscope with an LWD-20x air objective.

## Results

### Fluorescence-Based Sensor

Working with a simple epifluorescent light path and a dual-bandpass filter set, we developed a 3D-printable sensor that tracks the filtration status of a rudimentary glomerulus-on-a-chip system. This functions by quantification of two target molecules: inulin conjugated with fluorescein isothiocyanate (FITC), a sugar freely filtered by the glomerulus, and human-serum albumin (HSA) conjugated with Texas Red, a protein restricted in an intact glomerular barrier. The sensor system is designed for maximum accessibility and can be constructed out of recycled, or off-the-shelf and affordable, lab parts. In fact, the model used for the purposes of this paper heavily utilizes a recycled Thermo-Fisher® Countess II™ system (Online Resource 1A).

**Fig. 1:**
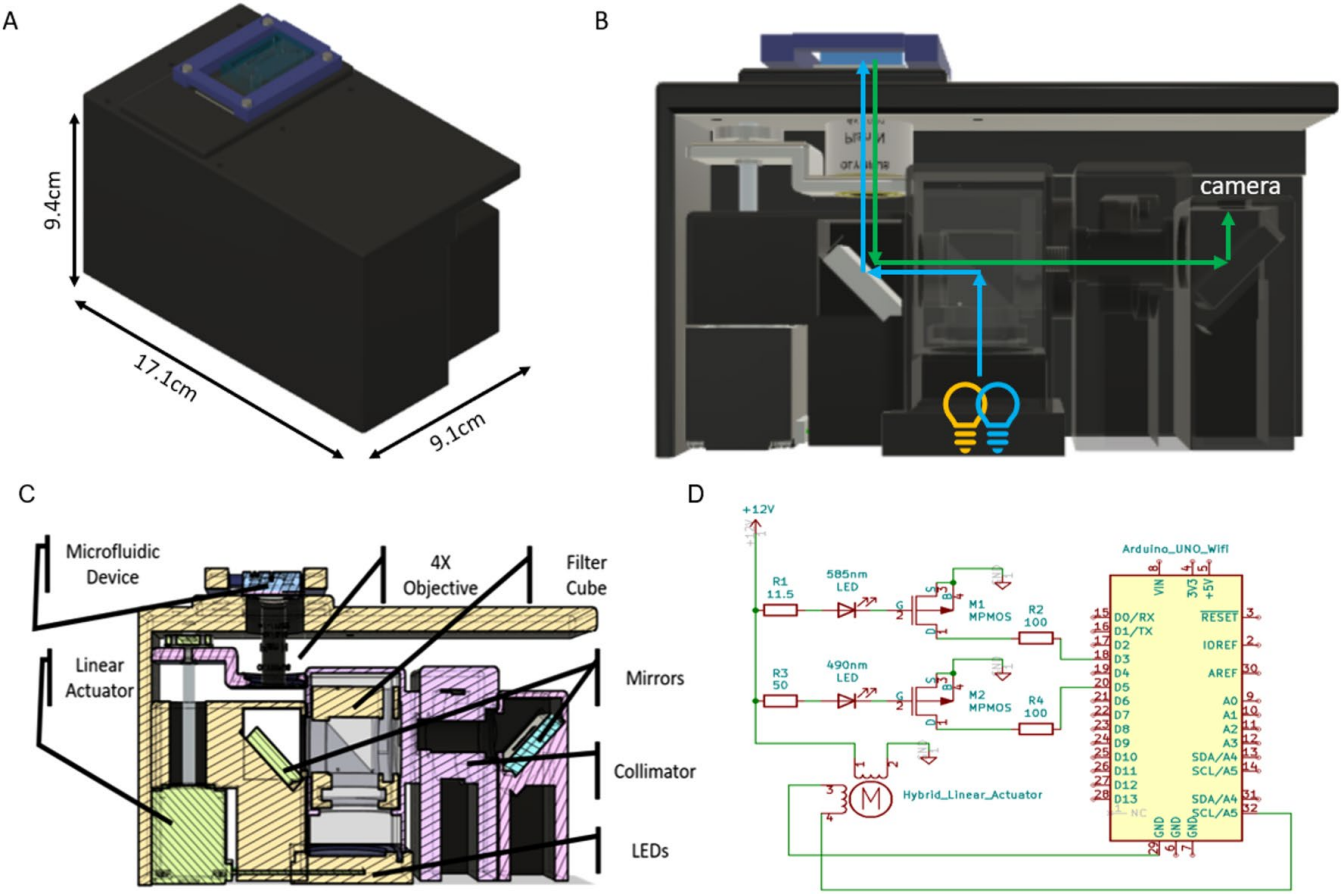
Three-dimensional rendering and electronic circuit diagram of the open-source sensor system. **A** Rendering of the 3D-printable fluorescence sensor, including dimensions and a rendered microfluidic device mounted atop the sensor. **B** Sketch of the light path for the excitation of the target molecules by high-powered LEDs (blue) and light path for the fluorescent emission of the target molecules (green) to be imaged by a Raspberry Pi camera and analyzed. **C** Cross-sectional view of the rendered open-sourced parts to build the fluorescence sensor, with individual parts labeled. **D** Circuit wiring diagram of the electronics for the fluorescence sensor, driven by an Arduino UNO, that control stage movement, LED on/off status and brightness via pulse width modulation

Design files for the completely 3D-printable light path, outlined in Fig. 1, are presented in the accompanying data repository (Zenodo, doi.org/10.5281/zenodo.7968008). Our laser-cut microfluidic device (Online Resource 2, Chip–Editable.f3z) was mounted and secured onto the stage using a 3D-printed buckle containing rare earth magnets (Online Resource 1B). We use a 0.1 NA 4X Plan Achromat (Olympus, Part #RMS4X) objective. A filter cube was 3D-printed (Online Resource 1C) to hold excitation and emission filters and a beam-splitter (Chroma, Part # 59010x, 59010 m, and 69015bs, respectively). This filter combination allows for simultaneous imaging of both FITC and Texas Red dye conjugates, which are bound to molecules with differing filtration efficiencies. The light path can be tailored with different combinations of filters to match the desired fluorophores. Our dual excitation source consists of a PCB with three amber (595 nm) LEDs (Luxeon-C, #J036-L1CAMB) compatible for Texas Red excitation and one cyan LED (490 nm) MCPCB LED (Thor Labs, #M490D3) compatible for FITC excitation. The LEDs were mounted in a 3D-printed LED holder (Online Resource 2, LED Holder.stl). The excitation and emission light paths are outlined in Fig. 1B. The entire light path was encased in laser-cut opaque black acrylic (Online Resource 2, Optical Path Casing.f3d), with ports cut for power leads and fluidics tubing and interlocking sides for easy assembly.

For ease of use, the sensor was automated using Raspberry Pi™ 4 Model B and Arduino UNO Wi-Fi. The vertical stage movement was automated with a linear actuator stepper motor (Haydon-Kerk, #E28H4-7-05-910), allowing for automated focus control. An Arduino controlled pulse width modulation circuit was designed to give user control over LED brightness and stage height (Fig. 1D). The LED illumination system and Arduino was powered by a 12 VDC, 1 A wall plug-in power supply (McMaster-Carr, Part # 70235K556). In our model, all parts were soldered onto a PCB to ensure stability, but the circuit can also be built on a breadboard. Arduino code was written in C++ and uploaded from a personal computer into the Arduino (Online Resource 2, Sensor.ino).

We developed a graphical user interface (GUI) in Python for imaging the chip that enables basic user control and live streaming of the field of view (Online Resource 3). The GUI was run on a Raspberry Pi™ single board computer (Supp. Table 1, sensor.py). Images were acquired using a Raspberry Pi™ HQ Camera set (Chicago Electronic Distributors, #CED-SC0261) to 8-bit depth with the IR filter removed at 507 × 380 pixel resolution, binned eight times relative to the initial camera resolution to improve signal quality.

### Chip Design

**Fig. 2:**
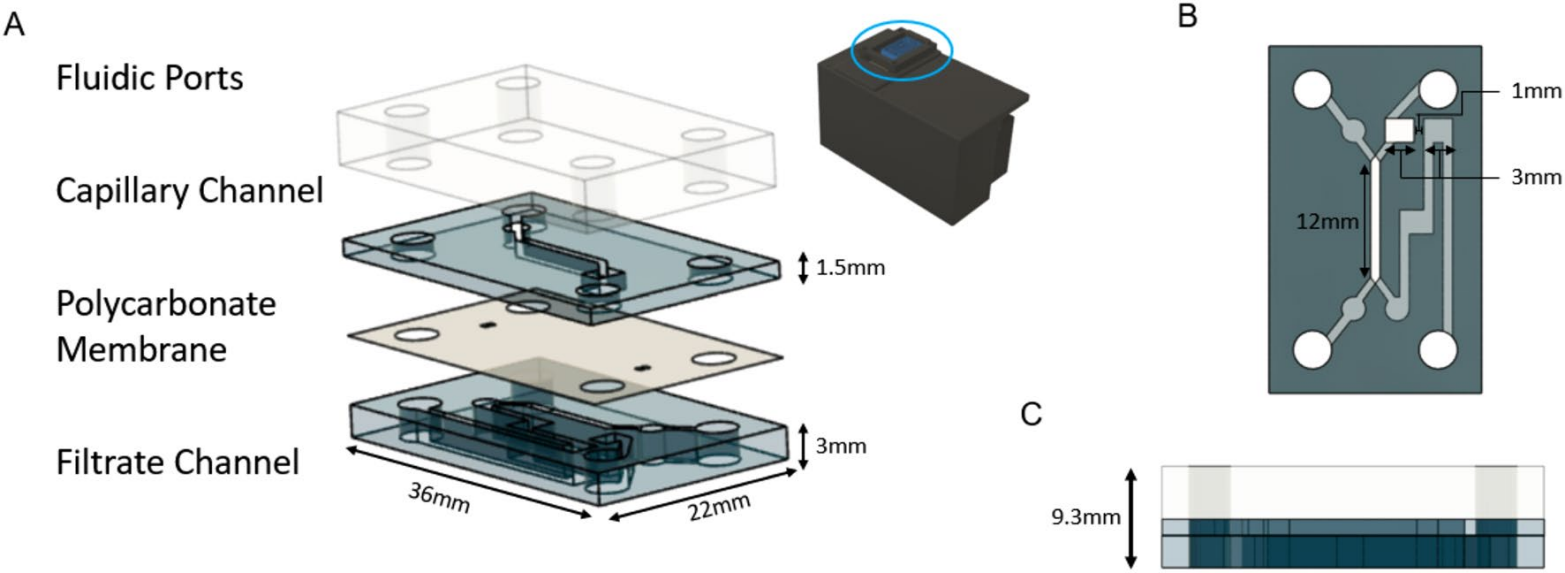
Microfluidic glomerular filtration barrier-on-chip microfluidic design. **A** Exploded view of PMMA-based microfluidic chip with macroscopic dimensions. The capillary channel faces endothelial cells, and the filtrate channel faces podocytes. The two channels are separated by a 20 µm track-etched polycarbonate membrane. **B** Top view of the PMMA-based microfluidic GFB-on-chip designed to enable simultaneous imaging of the capillary and filtrate channels with detailed dimensions. **C** Side view of the GFB-on-chip including fluidic port connections with macroscopic dimensions

A microfluidic chip was designed to model the human glomerulus as a test condition for the sensor system [21]. The chip consists of three laser-cut PMMA layers (Fig. 2A). The top layer faces endothelial cells and represents the capillary channel. The bottom layer faces podocytes and represents the filtrate channel. Atop the two channel layers is a thicker layer of PMMA that connects to the fluidic tubing. The two cellular layers are separated by a 20-µm-thick PCTE membrane with 1 µm pores.

The chip layout was designed so that both imaging channels can be acquired simultaneously from the same axial imaging plane (Fig. 2). The separation between the capillary (top fluidic segment) and filtrate (bottom fluidic segment) imaging squares is 1 mm and the field of view is 2 × 1 mm using a ×4 objective. Bubble traps are included at the fluidic inlets and outlets. Liquid from the capillary channel is imaged prior to crossing through the channel, passing through a trough to the bottom of the chip, where imaging can be performed from the bottom plane. Liquid from the filtrate channel is imaged after crossing through the channel.

### Sensor Calibration

**Fig. 3:**
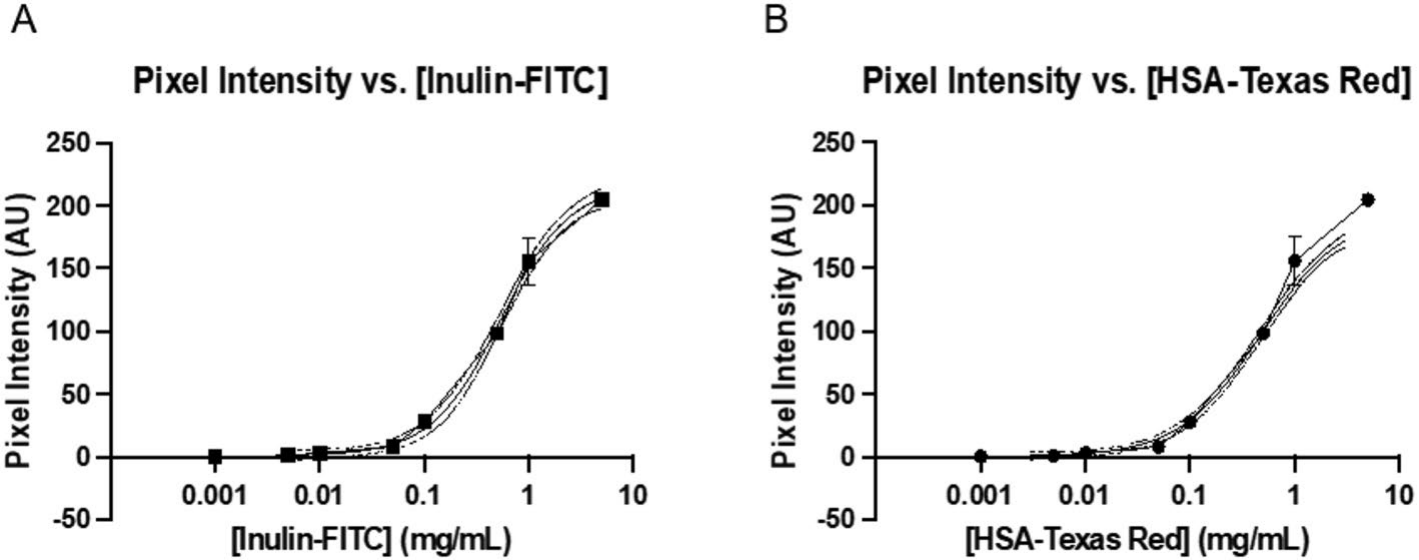
Calibration curves mapping mean pixel intensity value to target molecule concentration. Calibration curves on a semi-log scale for mean pixel intensity (8-bit depth arbitrary units, AU) value as a function of target molecule concentration for the desired target molecules across a clinically relevant range of (**A**) inulin-FITC and (**B**) HSA-Texas Red

We calibrated the sensor’s pixel intensity values to the target molecule concentration as the sensitivity of each sensor and target molecule-dye combination is unique. The pixel values collected were fit to a 4-paramater agonist vs. response curve using PRISM™ (Fig. 3). The sigmoidal response curve standardized the relationship between pixel intensity and the concentration of the target molecule, which can be used to determine the concentration of the target molecules based on the intensity values collected by the sensor. Based on the results of the non-linear regression, the top and bottom of the sensor’s sensitivity region for inulin-FITC detection were determined to be [11.2 ± 0.306 µg/mL, 4.39 ± 0.000306 mg/mL] (R^2^ = 0.9945). Similarly, the linear detection region for HSA-Texas Red concentration was found to be [25.7 ± 1.51 µg/mL, 4.426 ± 0.00151 mg/mL] (R^2^ = 0.9917). Though calibration is useful, to counteract potential variation in degree of labeling between batches, we found that filtration function is best analyzed by normalizing pixel intensity at the filtrate channel relative to pixel intensity at the capillary channel, thereby measuring diffusion percentage.

### Sensor Quality Control

**Fig. 4:**
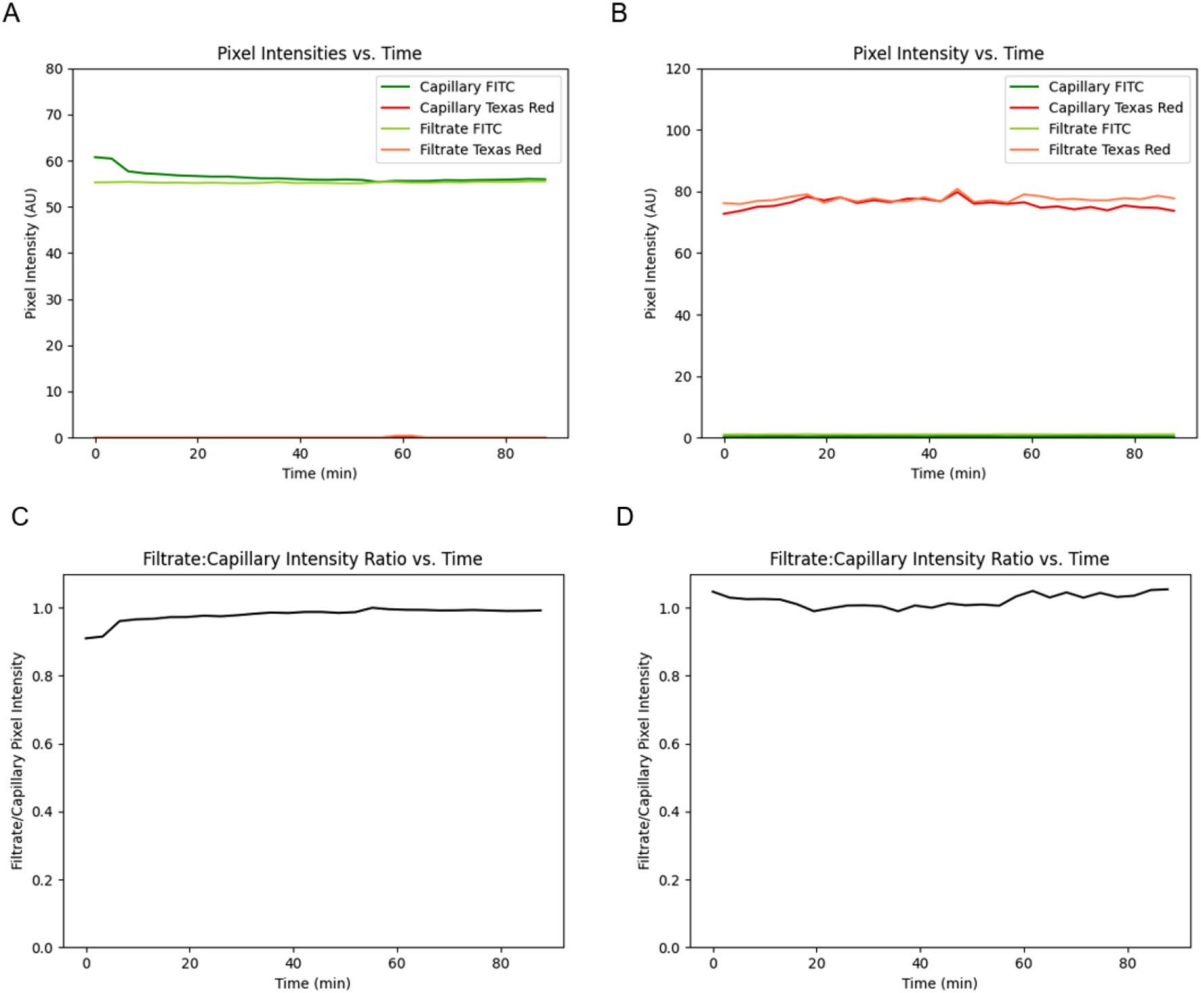
Sensor system stability over time. Readouts for the two measured channels quantified over time to evaluate system stability; **A** fluorescent signal intensities over time when the system is loaded with 0.5 mg/mL of inulin-FITC only on both sides; and **B** fluorescent signal intensities when the system is loaded with 0.5 mg/mL of HSA-Texas Red only on both sides. Ratio of mean intensity values between the filtrate and capillary sections of the chip, measured over time for **C** inulin-FITC only condition, as shown in Fig. 3A, and for **D** HSA-Texas Red only condition, as shown in Fig. 3B

We validated the illumination module by ensuring that only one target molecule was excited at each time (Online Resource 4). We did this by adding 20 µL of each target molecule conjugate at a concentration of 0.5 mg/mL in PBS to each imaging chamber. Each LED was selectively turned on and an image was acquired every 2 min for a total of 60 min (Fig. 4A, B). Across the 60-min period, we observed a stable signal. Additionally, when plotting the ratio of the signal at the filtrate channel to the signal at the capillary channel (Fig. 4C, D), we observed that the signals at each channel were essentially equal, as expected.

### Real-Time Quantification of Filtration

**Fig. 5:**
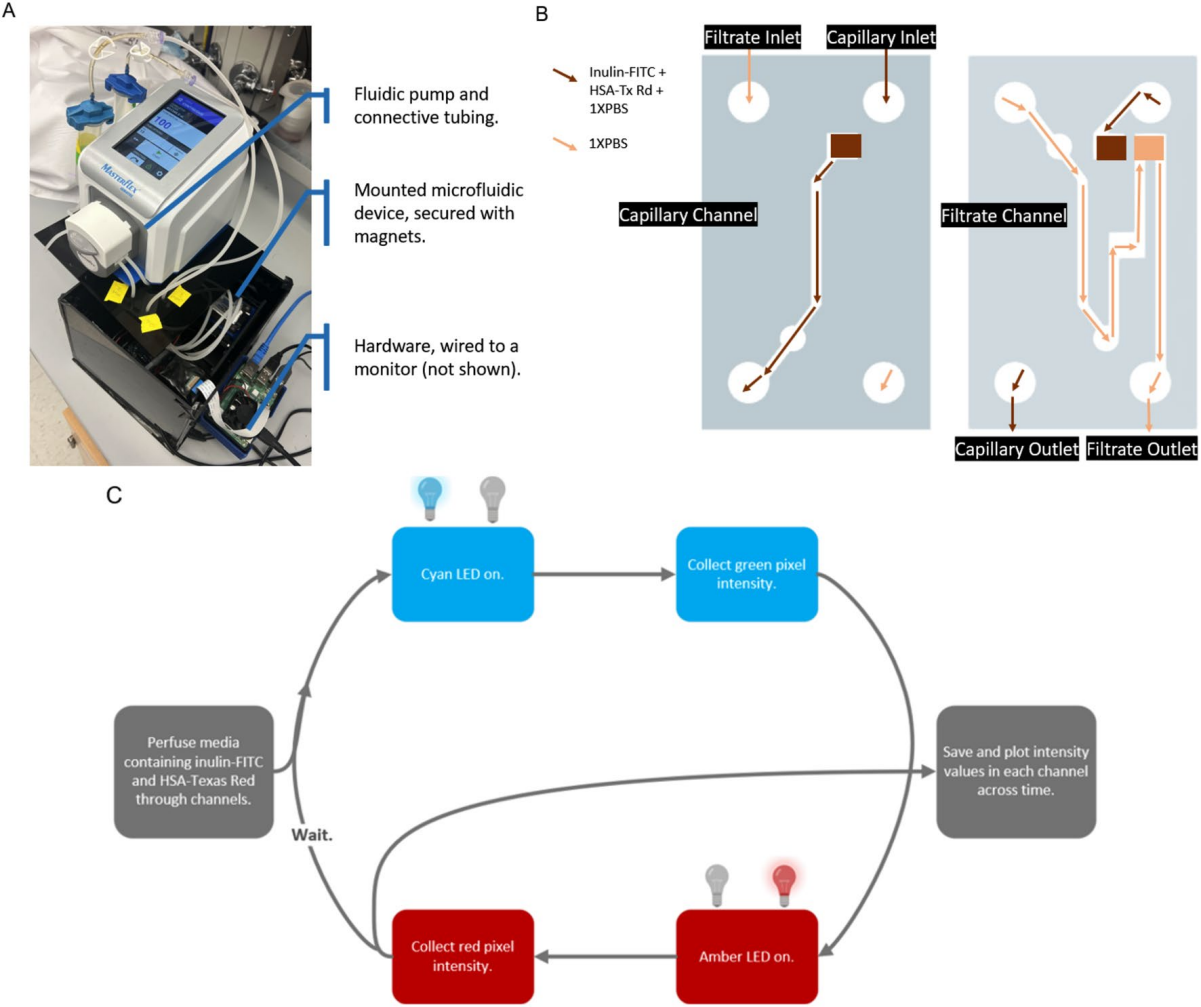
Experimental setup and flow diagrams for functional assessment of GFB-on-chip. **A** Setup of microfluidic GFB-on-chip and fluidic tubing for functional assessment using the fluorescence sensor. A fluidic pump is mounted atop the acrylic sensor casing. **B** Path of fluid flow through the GFB-on-chip used for functional assessment of the filtration barrier. **C** Flowchart of the Python-driven automated data collection cycle for real-time functional assessment of the GFB-on-chip using the fluorescence sensor

To quantify the real-time filtration status of the organ-on-chip model, our microfluidic device was centered on the sensor’s field of view and our fluidic pump was placed atop the sensor’s acrylic enclosure (Fig. 5A). Regions of interest (ROIs) were selected in the software to indicate the capillary and filtrate channels, which are on the left and right of the field of view, respectively (Fig. 5B). ROIs were selected using the entire vertical range of the field of view. A background image was collected with a media blank to correct for potential light leakage. Next, the automated data collection cycle was initiated (Fig. 5C), and pixel intensities were collected across a user-specified length of time.

Throughout the user-specified length of time, HSA-Texas Red and inulin-FITC in PBS were perfused into the capillary channel via the fluidic port. 1× PBS was perfused through the filtrate channel anti-parallel to the capillary channel (Fig. 5B). Both channels were perfused in a closed loop over the user defined imaging period. Over a user-specified length of time, the sensor quantified concentration in the capillary and filtrate channels via the process mapped in Fig. 5C. Background subtraction was performed prior to quantification. Data were acquired with 2-min intervals. The comparison of pixel intensity values between the two ROIs across time allowed for continuous evaluation of the functional status of the filtration barrier. The tracking of inulin-FITC and HSA-Texas Red is shown in this report, and the sensor can be adapted to evaluate any two target molecules across a selective filtration barrier by seeding different cell types and conjugating different molecules (Fig. 5).

**Fig. 6:**
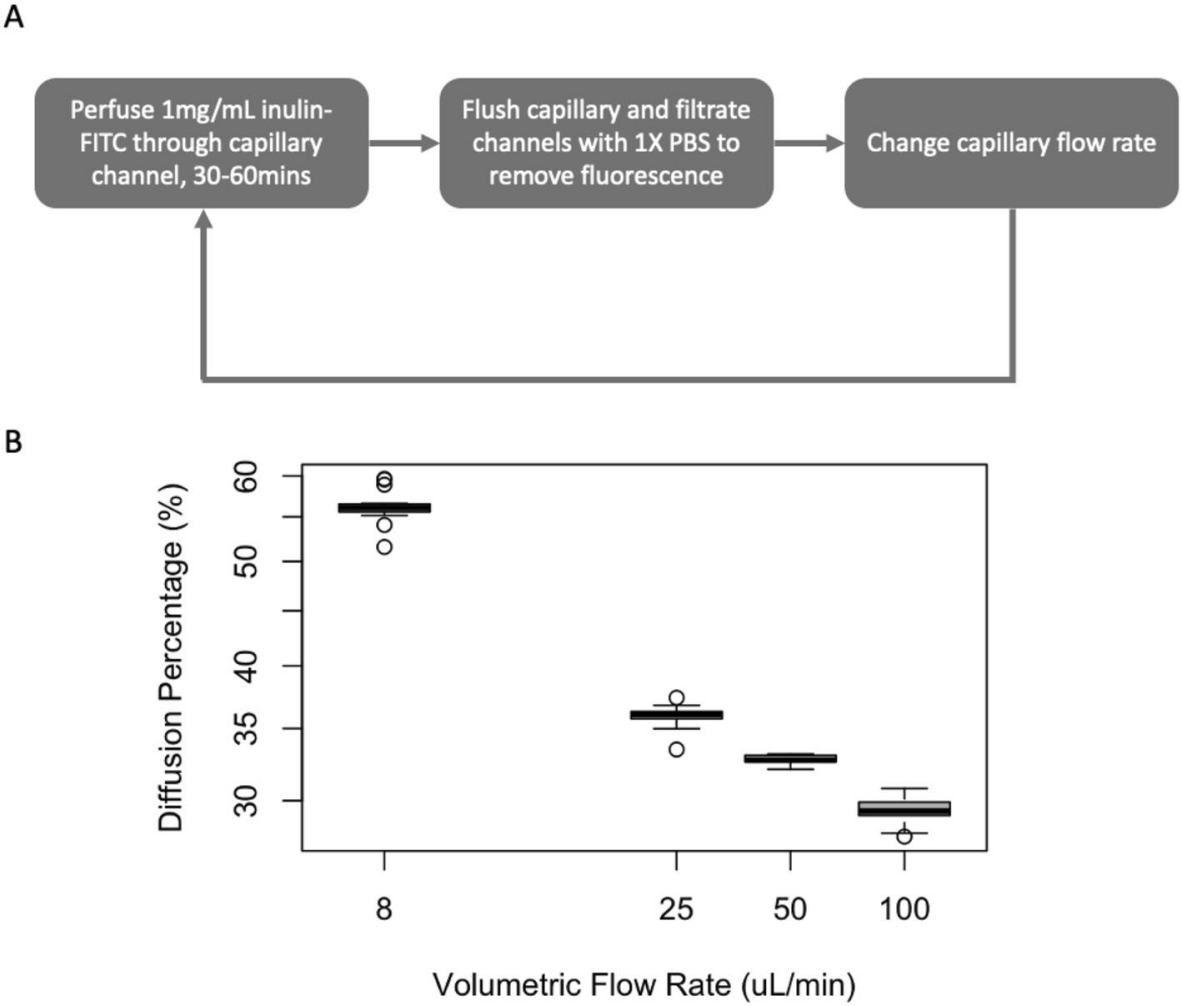
Detection of changes in steady-state diffusion percentages due to changing volumetric flowrates. **A** Experimental workflow used to evaluate effects of changes in volumetric flowrate on diffusion percentage, using the fluorescence sensor in a cell-free context. **B** Steady-state diffusion percentages at different flowrates

We utilized a cell-free context to demonstrate the sensor’s detection of changes in the steady-state diffusion percentage at various volumetric flowrates. Starting with a perfusion rate of 100 µL/min, and following the experimental procedure illustrated in Fig. 6A, flowrate was decreased incrementally to 50 µL/min, 25 µL/min, and 8 µL/min. Wall shear stress at the membrane surface for these conditions were 30.7, 15.4, 7.68 and 2.46 x 10-3 dynes/cm^2^, respectively, calculated using Eq. (3). The diffusion percentage indicates how much inulin and albumin cross through the membrane at any given flow velocity. Given the setup’s cell-free context, the increase in shear stress will have limited impact on the diffusion as there are no cellular changes to impact barrier permeability. Thus, based on fluid dynamics within the microfluidic device, as outlined in equations 2, 4–11, the predicted equilibrium diffusion percentages for the volumetric flowrates of 8, 25, 50, 100 µL/min would decrease as the flowrate increases. The sensor-quantified diffusion percentages at equilibrium for the different flowrates are shown in Fig. 6B, with flowrate on a log2 scale. With increasing flowrates, we see decreasing steady-state diffusion, in line with mathematical prediction in a cell-free context. Our sensor reflects these predictions, and as the flowrate decreases, the steady-state percentage of free diffusion increases (Fig. 6B).

**Fig. 7:**
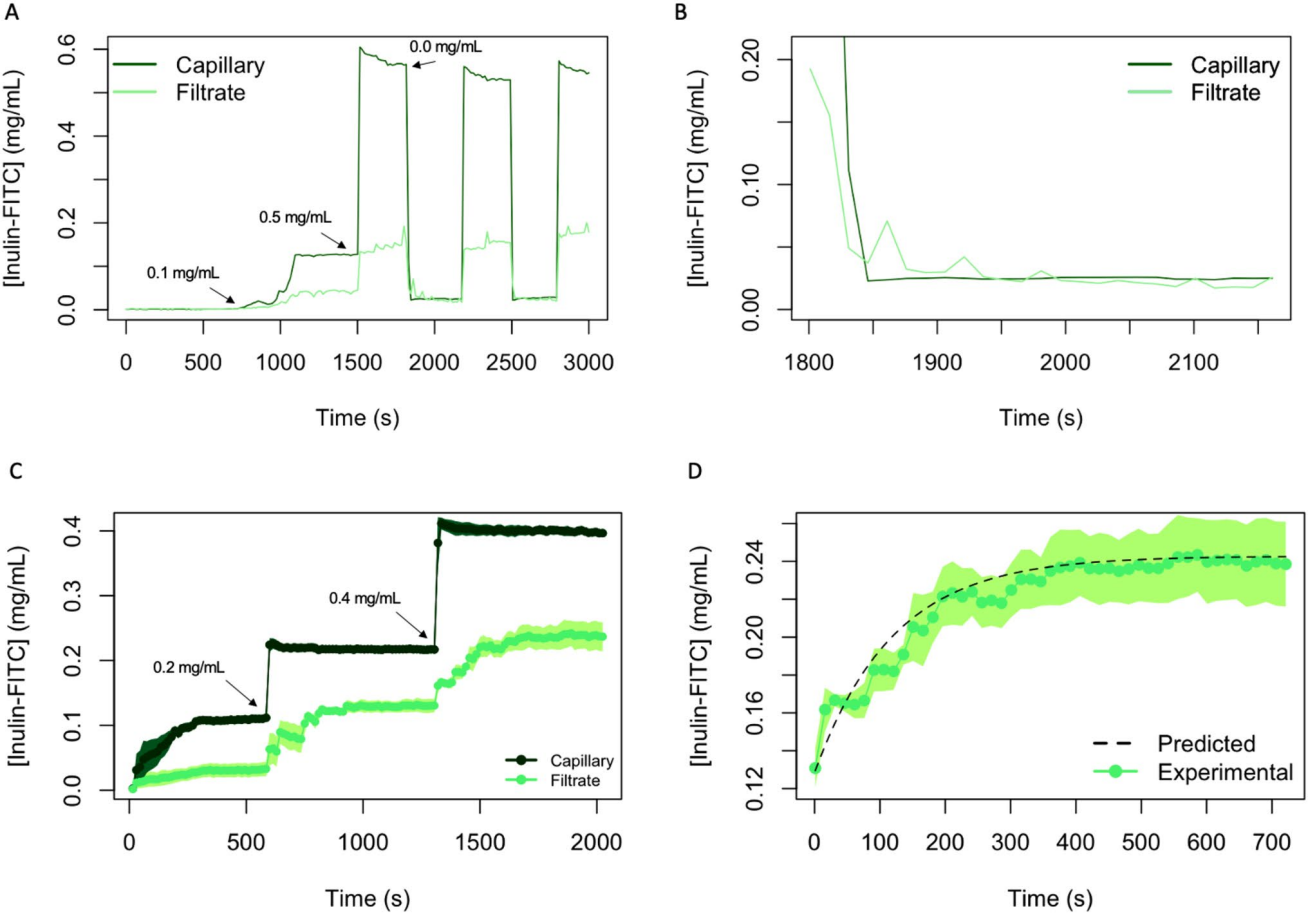
Real-time visualization of diffusion percentage under different concentration gradients. **A** Real-time sensor readout in response to changing capillary inulin-FITC concentration from 0.1 to 0.5 to 0.0 mg/mL repeated three times. **B** Zoomed-in view of real-time decrease in filtrate inulin-FITC concentration from the first 0.5 to 0.0 mg/mL capillary concentration change. **C** Real-time sensor readout in response to changing capillary inulin-FITC concentration from 0.1 to 0.2 to 0.4 mg/mL. **D** Filtrate concentration for last step (from 0.2 to 0.4 mg/mL in the capillary channel) and comparative theoretical prediction for filtrate concentration transient response to the gradient change

To assess the real-time capabilities of the sensor, varying magnitudes of concentration gradients were applied to the microfluidic device; fluorescent sensor system and automated quantification were simultaneously operated, and dynamic changes in diffusion were observed in real time. Results show that the filtrate concentration drops with concentration drops in the capillary channel (Fig. 7A). Additionally, by viewing the filtrate concentration behavior on a zoomed-in view, it is possible to see the time-dependent decrease of concentration which approaches the equilibrium point of the capillary channel (Fig. 7B). To examine the dynamic behavior of the system more closely, concentration gradient was increased in stepwise manner by injecting 0.1, 0.2, and 0.4 mg/mL inulin-FITC into the capillary segment. The average sensor-quantified concentration values from three independent trials were the plotted (Fig. 7C). Without diluting either channel segment, a more accurate prediction of equilibrium concentrations could be made. Based on Eqs. (4)–(11), it was predicted that the filtrate equilibrium concentration that correspond to capillary channel concentrations of 0.1, 0.2, and 0.4 mg/mL would be 0.0344, 0.18, and 0.24 mg/mL, respectively. Results showed that the target molecule concentrations in the filtrate channel followed the changes in the driving concentration gradient as expected. When the third filtrate step response (i.e., moving capillary concentration from 0.2 to 0.4 mg/mL) was plotted along with the theoretical dynamic response of the same step, as estimated in Eq. (12), the experimental and theoretical gradient responses matched closely (Fig. 7D) [30].

### Cellularized Glomerulus-On-Chip

**Fig. 8:**
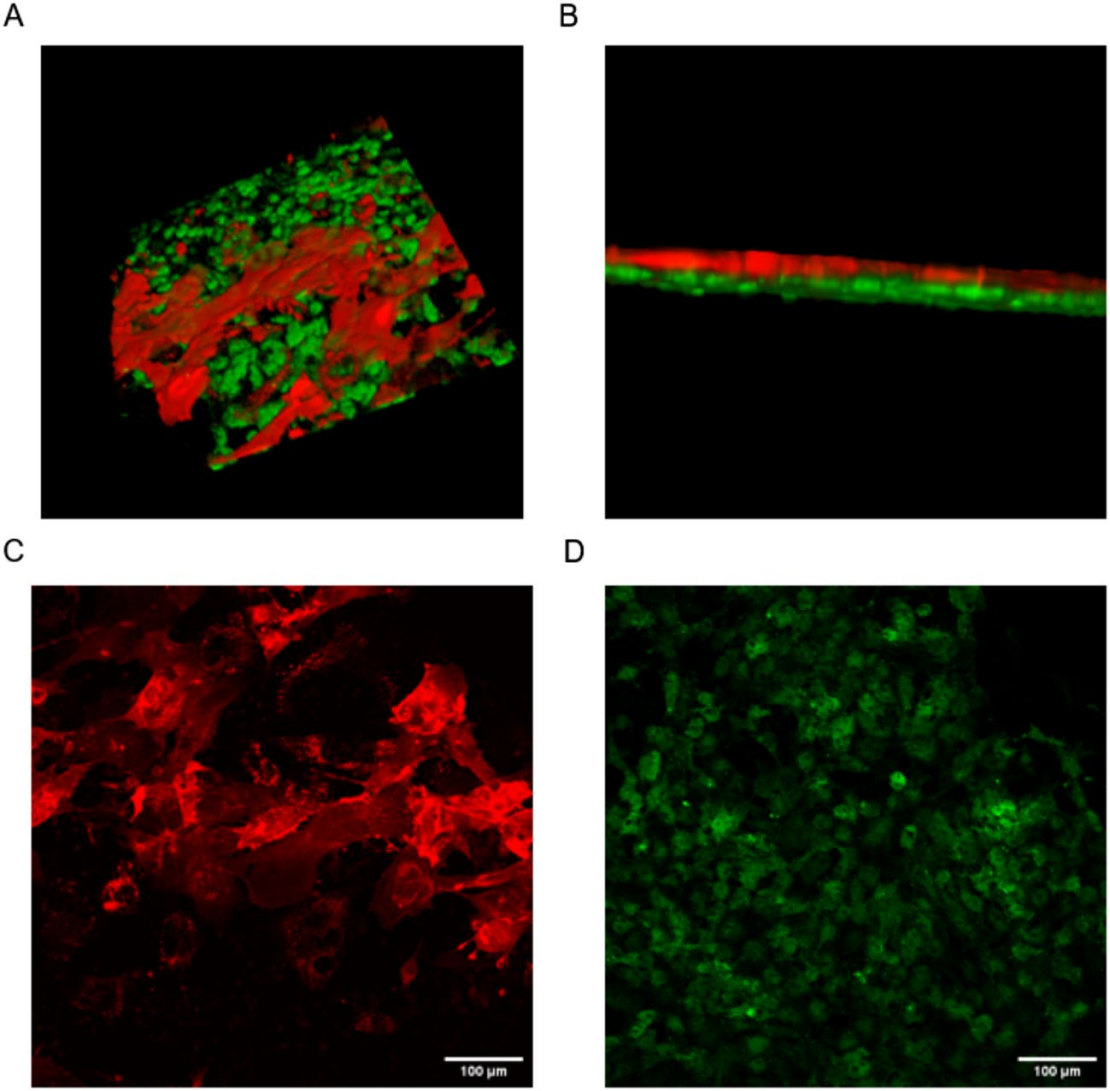
Multicellular filtration layer forming the complete glomerulus-on-chip. Two cell types were seeded on-chip: differentiated human podocytes that stably express mScarlet-LifeAct (red) and HUVECs that stably express mClover-LifeAct (green). The multicellularized filtration barrier is visualized on-chip using laser scanning confocal microscopy. Maximum intensity projection of 3D reconstructed volume is shown from the (**A**) oblique perspective and **B** side view. **C** Visualization of only the podocyte layer, and **D** visualization of only the HUVEC layer

To ensure OOC integrability, two cellular monolayers were grown on the microfluidic chip as a proof-of-principle experiment. Following previously established protocols [7, 21], we demonstrate the ability to culture a cellular filtration barrier, akin to a glomerulus-on-a-chip, using the PMMA and the cut-tape design. Immortalized podocytes that stably express mScarlet-tagged LifeAct were seeded and grown on-chip in the filtrate channel while HUVECs that stably express mClover-tagged LifeAct were seeded in the capillary channel after fibronectin coating. We show that the polycarbonate membrane enables co-culture of two different cell types (Fig. 8A) and maintains separation between the two cellular layers (Fig. 8). We note that extensive projection formation for podocytes across the channel region were observed, forming primitive cell–cell interactions (Fig. 8C, D). These experiments show that the sensor can be integrated with a glomerulus-on-chip for real-time sensing of the integrity of the cellular monolayers.

## Discussion

We present an economical system for real-time, quantitative, noninvasive assessment of filtration status in engineered microphysiological systems using off-the-shelf components. The final prototype, which is available through a CC-BY 4.0 open-access license, is intended to improve accessibility of engineered systems to the greater cell biology community. Through this economical, open-source assay, research centers with non-engineering expertise can expand their capabilities into OOC applications, e.g., quantitative functional assessment of patient-derived kidney cells across time. Completely open-sourced, using parts readily obtainable across the world, the cost of constructing the sensor system, at the time of this publication, is comparable to purchasing three research-grade antibodies (Online Resource 5). We also note the proposed chip fabrication process does not require usage of a cleanroom or extensive personal protective equipment, such as masks and/or gowns. Because the fabrication does not require a specialized facility, the process is cheaper and more accessible than many alternatives. Gloves were used while applying sealant for extra precaution; however, all materials used are nontoxic. Additionally, the proposed design is applicable for any two target molecules by use of the included protocol to conjugate FITC and Texas Red dyes (or by choosing other filter combinations that will enable imaging of desired fluorescent molecules).

One advantage of this approach is the ability to fluorescently label any marker of interest [37]. Additionally, with appropriate calibration of the background signature and dye response, the system could allow the incorporation of different solvents, such as human blood, as long as the molecular diameter of interest remains smaller than the pore of the PCTE membrane. Beyond assembly and functional assessment, large-scale applicability of OOC systems requires accessible parts and relative affordability. Currently, there is a lack of open-source OOC systems and evaluation hardware available for easy construction, which can be used to assess real-time function of patient-derived engineered tissue systems. To address this, we have developed and evaluated a non-invasive fluorescence-based system for tracking real-time selective filtration of two key kidney health biomarkers: human-serum albumin (HSA) and inulin.

In the cell-free context that was tested, our sensor system can linearly detect concentrations of inulin-FITC from 0.0112 to 4.39 mg/mL and HSA-Texas Red from 0.0257 to 4.426 mg/mL, which are ranges clinically relevant to kidney dysfunction [38]. We further demonstrate the sensor’s capabilities in real-time diffusion sensing and system response to dynamic changes in concentration gradients within a microfluidic device. Time-dependent changes in diffusion demonstrated by the sensor align with previously published simulated and experimental fluid dynamics [30, 35]. Even though these dynamic experiments were carried out in the absence of renal cell lines, the system was demonstrably designed to support cellularization. Our proof-of-principle studies with immortalized human podocyte and endothelial cell lines, respectively, showed arborization and monolayer formation behaviors similar to those observed in other OOC systems [7]. While it is the outside the scope of this open-access engineering study, future studies using this system will focus on cellular function and response, such as distribution of slit diaphragm proteins within the system.

There are several limitations to our device that should be noted. Any concentration lower than the levels within the sensor’s linear sensing regions may not be accurately probed by the sensor, which may preclude the use of the device for assays that require a more precise concentration reading. However, if necessary, this limitation could be addressed by using brighter fluorescent probes; we opted to use the current probes as they are the most widely available and economical ones. Another limitation of the system is that it is currently designed for applications that require two target molecules, as adding a third tracked molecule would require modification of the light path (e.g., addition of LEDs and/or filter sets). We also acknowledge that our present OOC model does not facilitate significant levels of diffusion at 100 µL/min due to its low-pressure differential and small channel area; however, this limitation can be overcome by reducing the channel thicknesses. We deliberately selected these dimensions to maximize general adaptability of the instrument, as thinner channels (which would exponentially increase rates of diffusion) would either require a nanofabrication facility or prohibitive upfront material costs. A balance was sought to maintain proper levels of shear stress on the cellular layers, which restricts the minimum flowrate usable in the current setup. While we acknowledge these limitations, we are actively exploring ways to inexpensively improve the precision of the sensor and building a microphysiological fluidic system that enables levels of filtration closer to those in vivo. Our approach enables real-time tracking of the filtration status without manual extraction of media from active perfusion microfluidic systems, which could improve previously reported glomerulus-on-chip systems that require manual intervention [7]. Minimizing manual intervention allows decreased experimental variability thus allowing visualization of changes in filtration status caused by drugs (rather than potential interference due to extraction of media from the system). At 22 × 20 × 18 cm, the entire system can fit into a standard-sized incubator, allowing long-term monitoring. Finally, even though our relatively simple system requires introduction of exogenous dyes into the circulation, it operates at or near real time, and the dyes can easily be washed or diluted out. Our seven-second time resolution is significantly lower than manual or automated systems, such as the 58 min reported for electrochemical monitoring [39]. Real-time testing of the system at even a fifteen second sampling period demonstrates the additional information gained from this increased time resolution.

In conclusion, we present an affordable and open-source assay that can be broadly used for applications requiring real-time tracking of cellular filtration status of microphysiological systems. With minor modifications, this system can be adapted to many other OOC applications that require live tracking of molecular species across semipermeable membranes, such as gut, liver, blood–brain barrier, or lung microphysiological systems. We demonstrate integrability with multiple cellular layers, which are central for OOC applications. Lastly, we present a detailed protocol for setting up and operating the system with minimal technical background. The simplicity of our design, its relative affordability, detailed protocols, and open-sourced design files will provide the ability to increase the throughput of many precision medicine assays, such as nephrotoxicity screening using patient-derived cells and/or drug testing [40].

### Supplementary Information

Below is the link to the electronic supplementary material.

Electronic supplementary material 1 (PDF 836 kb)

## Data Availability

All code and downloadable design files can be located at https://github.com/AzelogluLab/Sensor. All raw data and images used in this manuscript can be found on Zenodo at https://doi.org/10.5281/zenodo.7968008.
