# Supplementary material for: Open-Source System for Real-Time Functional Assessment of In Vitro Filtration Barriers
Source: Ann Biomed Eng. 2023 Oct 29;52(2):327–41. doi: 10.1007/s10439-023-03378-9 (PMC10808466; doi:10.1007/s10439-023-03378-9)
Supplement: Supplementary file 1 — Electronic supplementary material 1 (PDF 836 kb) [file 10439_2023_3378_MOESM1_ESM.pdf]

OPEN-SOURCE SYSTEM FOR REAL-TIME FUNCTIONAL ASSESSMENT OF IN VITRO FILTRATION BARRIERS

Tess K. Fallon (1), Merve Zuvin (1)*, Alan D. Stern (1)*, Nanditha Anandakrishnan (1), Ilse S. Daehn (1), Evren U. Azeloglu (1,2)

(1) Barbara T. Murphy Division of Nephrology, Icahn School of Medicine at Mount Sinai, New York, NY, USA
(2) Pharmacological Sciences, Icahn School of Medicine at Mount Sinai, New York, NY, USA

*Indicates equal contribution.

Address for Correspondence:

Evren U. Azeloglu, Ph.D.
Associate Professor of Medicine, Division of Nephrology
Associate Professor of Pharmacological Sciences
Icahn School of Medicine at Mount Sinai
One Gustave L. Levy Place, Box 1243
New York, NY 10029

Email: evren.azeloglu@mssm.edu
Voice: 212-241-8519
Twitter: @azeloglu

Online Resource 1. Images and 3-D render of individual sensor parts. (A) Image and trace of light path and sensor used, using a retrofitted Thermo-Fisher© Countess II. **(B)** Image of the microfluidic device mounted atop the sensor, secured with rare-earth magnets. **(C)** Rendering of the filter cube used to mount the optical filter set used for emission and excitation of target molecules, inulin-FITC and HSA-Texas Red.

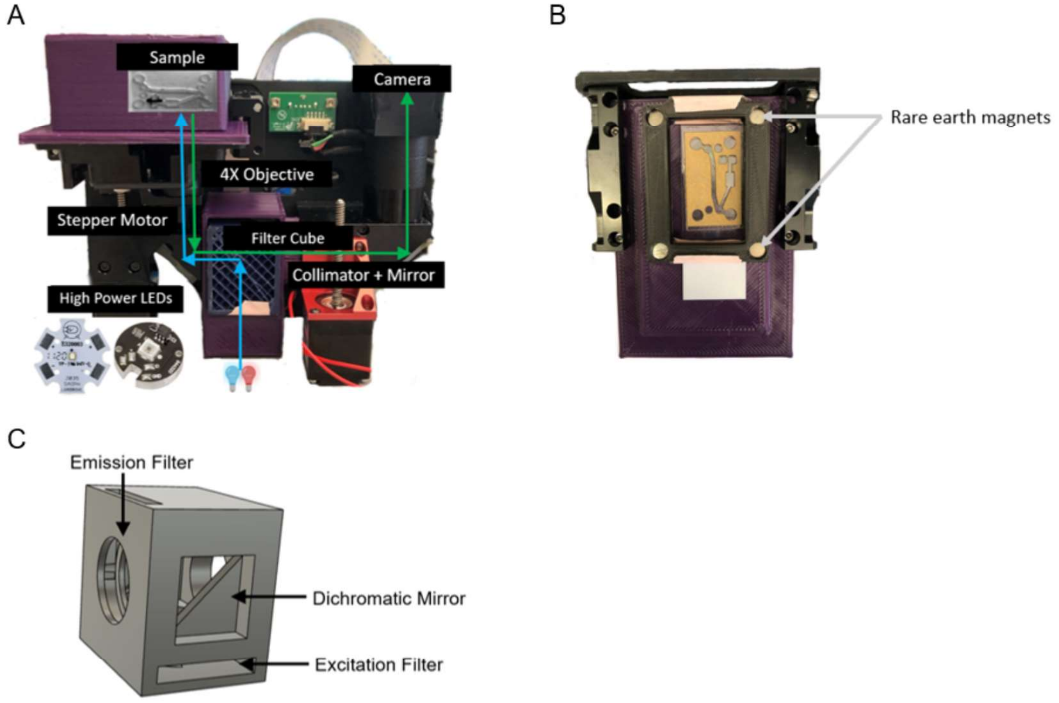

Online Resource 2. Table describing all open-source files which can be used to build and use the sensor platform. All design files can be found at: https://github.com/AzelogluLab/Sensor

Filename	Description
/3D Printable Parts/Optical Path/Optical Path.f3z	Editable project for the entire optical path, openable in Fusion 360.
/3D Printable Parts/Optical Path/Chip Clamp.stl	3D-printable .stl file for the magnetic clamp, which secures the microfluidic device onto the stage.
/3D Printable Parts/Optical Path/Collimator Holder.stl	3D-printable .stl file designed to hold the optical collimator linked in Supp. Information Table 1.
/3D Printable Parts/Optical Path/Filter Cube Holder.stl	3D-printable .stl file designed to hold the custom filter cube.
/3D Printable Parts/Optical Path/Filter Cube.stl	3D-printable .stl file designed to hold the filter combination linked in Supp. Information Table 1. Includes slots for filters and built-in support for dichromic mirror positioning at 45°.
/3D Printable Parts/Optical Path/LED Holder.stl	3D-printable .stl file designed to hold the LEDs linked in Supp. Information Table 1. Indents for friction hold of LEDs included, but super glue also recommended for maximum LED stability.
/3D Printable Parts/Optical Path/Mirror Holder With Camera Mount.stl	3D-printable .stl file designed to hold the optical mirror linked in Supp. Information Table and mount the Raspberry Pi HQ Camera.
/3D Printable Parts/Optical Path/Mirror Holder.stl	3D-printable .stl file designed to hold the optical mirror linked in Supp. Information Table 1.
/3D Printable Parts/Optical Path/Objective Holder.stl	3D-printable .stl file designed to hold the objective linked in Supp. Information Table 1 and to integrate with the linear actuator linked in Supp. Information Table 1.
/Laser Cuttable Parts/Optical Path Casing	Contains .dxf files and an editable .f3d file for the acrylic casing, including holes to enable access to power, fluidic tubing, and an external monitor.
/Laser Cuttable Parts /Chip/Capillary Channel – Top.dxf	Laser-cuttable .dxf file in mm scale for the capillary (top) channel of microfluidic glomerulus-on-chip. Designed for simultaneous imaging of capillary and filtrate channels.
/Laser Cuttable Parts /Chip/Filtrate Channel – Bottom.dxf	Laser-cuttable .dxf file in mm scale for the filtrate (bottom) channel of microfluidic glomerulus-on-chip. Designed for simultaneous imaging of capillary and filtrate channels.
/Laser Cuttable Parts/Chip/Fluidic Port Connection.dxf	Laser-cuttable .dxf file in mm scale for the top most piece of the microfluidic glomerulus-on-chip used to connect to the fluidic ports. Requires tapping with a manual tap to generate the threading.
/Code/Sensor.ino	Script used to control Arduino operations, LED brightness, and stage movement via stepper motor. Must be uploaded to the Arduino prior to running the assay via USB port.

/Code/sensor.py	Script used to automate data collection and enable manual control of sensor. Integrates with Raspberry Pi. Includes real-time camera visualization, image stabilization, and graphical user interface. Prior to use, must run commands outlined in Sensor/README.md.

Online Resource 3. Graphical user interface used to automate data collection and simplify user experience.

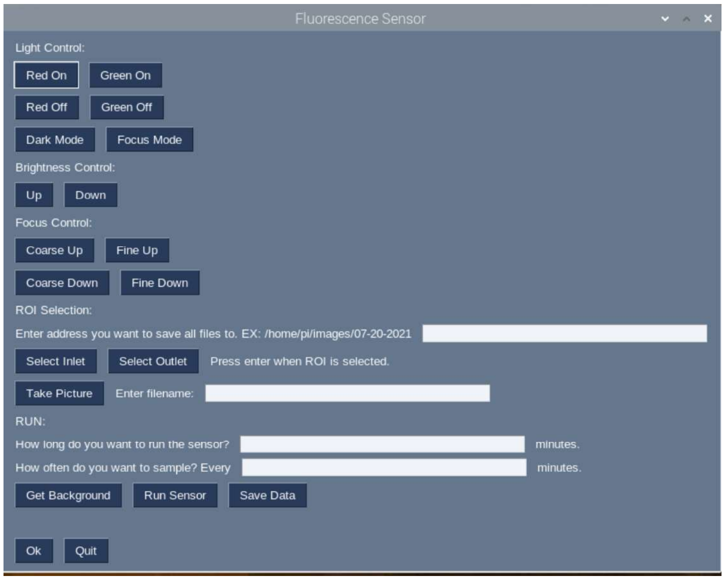

Online Resource 4. Illumination quality control for LED excitation of target molecules using 20 µL of inulin-FITC in the capillary (left) channel and 20 µL of HSA-Texas Red in the filtrate (right) channel. (A) Image of the imaging squares with both cyan and amber LEDs on**. (B)** Image of the imaging squares with both LEDs off**. (C)** Image of the imaging squares with only the cyan LED on, and the amber LED off**. (C)** Image of the imaging squares with only the amber LED on, and the cyan LED off**.**

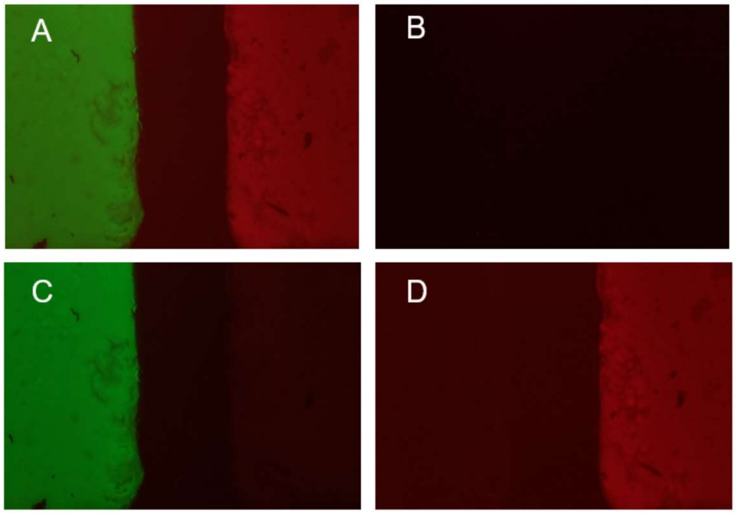

Online Resource 5. Table estimating cost of every part necessary to build the platform and links to where parts can be purchased.

**Component**<br><br>Optical<br>Electronic<br>Chemical	**Estimated Price (USD)**	**Purchasable At**
Mirror (2X)	77 (x2) = 154	https://www.thorlabs.com/thorproduct.cfm?partnumber=BBSQ1-E02
Achromatic Collimator	225	https://www.thorlabs.com/thorproduct.cfm?partnumber=F950FC-A
Dual Band Barrier Filter	350	https://www.chroma.com/products/parts/59010m
Dual Band Excitation Filter	350	https://www.chroma.com/products/parts/59010x
Dual Band Dichroic Beam-Splitter	375	https://www.chroma.com/products/parts/69015bs
Arduino UNO Starter Kit	36	https://www.amazon.com/ELEGOO-Project-Tutorial-Controller-Projects/dp/B01D8KOZF4/ref=sr_1_1_sspa?keywords=arduino+uno+starter+kit&qid=1684618593&s=industrial&sr=1-1-spons&psc=1&spLa=ZW5jcnlwdGVkUXVhbGlmaWVyPUEzVDFSU1c2VldCQjZVJmVuY3J5cHRlZElkPUEwOTAxMjEwMzRPSlREVFNENVlBJmVuY3J5cHRlZEFkSWQ9QTA5MTUwNjYzOEtZWFA5V0w5U1VCJndpZGdldE5hbWU9c3BfYXRmJmFjdGlvbj1jbGlja1JlZGlyZWN0JmRvTm90TG9nQ2xpY2s9dHJ1ZQ==
Raspberry Pi	35	https://www.adafruit.com/product/4295?src=raspberrypi
Raspberry Pi HQ Camera	50	https://www.raspberrypi.com/products/raspberry-pi-high-quality-camera/
USB Cord	5	https://www.digikey.com/en/products/detail/tripp-lite/U021-003/1533764
Linear Actuator	25	https://www.adafruit.com/product/5117
Motor Controller	22	https://www.adafruit.com/product/2348
12V AC/DC Converter	11	https://www.amazon.com/Adapter-100-240V-Transformers-SwitchingAdaptor/dp/B07GRZB5Y9/ref=asc_df_B07GRZB5Y9?tag=bingshoppinga20&linkCode=df0&hvadid=80814156749445&hvnetw=o&hvqmt=e&hvbmt=be&hvdev=c

		&hvlocint=&hvlocphy=&hvtargid=pla-4584413735555703&psc=1
LED (585nm)	8	https://www.ledsupply.com/leds/luxeon-c-color-leds
LED (490nm)	84	https://www.thorlabs.com/thorproduct.cfm?partnumber=M490D3
Inulin-FITC	526/g	https://www.sigmaaldrich.com/US/en/product/sigma/f3272
Human-Serum Albumin	86/g	https://www.sigmaaldrich.com/US/en/product/sigma/a1653
Texas Red Dye	377/5mg	https://www.thermofisher.com/order/catalog/product/T6134

TOTAL COST: $1730*
(*calculated using a per assay estimation for the target molecule and their conjugates)
